# The Importance of a Thorough Knowledge of Biology, Bacteriology and the Circulation of the Blood for the Successful Application of Serum Therapy

**Published:** 1908-11

**Authors:** J. C. Grady

**Affiliations:** Kenly, N. C.


### THE IMPORTANCE OF A THOROUGH KNOWLEDGE OF BIOLOGY, BACTERIOLOGY AND THE CIRCULATION OF THE BLOOD FOR THE SUCCESSFUL APPLICATION OF SERUM THERAPY.*

BY J. C. GRADY, M. D., KENLY, N. C.

The subject I have selected for my paper is supposedly a physiological one: "The Study of the Circulation of the Blood Biology and Bacteriology and Their Relation to Serum Therapy."

* Read before Medical Society, N. C., June 15, 1908.

But should I digress somewhat from the letter of my text, I beg your pardon and kind indulgence in advance, while I attempt to rehash this old threadbare subject that circulation of the blood is so inseperably connected with bacteriology and serum therapy. I do not expect to be able to advance any new ideas along these lines; but if I can succeed in provoking a discussion of the subject, then the object of my paper will be attained.

The uses of the circulating blood may be summarized thus: It is a medium for the reception and storing of matter, that is, oxygen and digested food materials from the outer world for conveyance to all parts of the body. It is also a source from which all the various tissues of the body may take the materials necessary for their nutrition and maintainance, and whence the secreting organs obtain the constituents of their various secretions.

It is also a medium for the absorption of deleterious or refuse matters from the various tissues and their conveyance to the eliminating organs for their expulsion lest the system become, by auto intoxication, her own destroyer.

It seems to me that a thorough and concise knowledge of the chemical constituents, biological elements, physiological and opsonic functions of the blood together with its course and manner of travel over the system is absolutely indispensible and merits our most thorough and painstaking study, if we would elucidate the mysteries and solve the problem of serum therapy and understand the important role the circulation plays in physiological and pathological processes. As you know, for the last few years there has been a great tidal wave of chemical and bacteriological research sweeping over this country and Europe. The human blood has been subjected to an endless variety of the most critical and searching tests, and this bacteriological crusade has put scientists and medical men everywhere, on the alert hunting for a more satisfactory aetiology and a more dependable treatment for the ills that afflict humanity. Medical and scientific men have been standing with microscope and chemical retort in hand striving to recognize and capture the baneful micro-organisms or materies morbi that foster disease and engender death, and while as yet the goal has not been attained, many new and startling truths have been evolved, many false and erroneous theories exploded, and many valuable improvements added to our rapidly increasing knowledge of bacteriology and serum therapy. Many of these innovations and improvements are things too that only a short while go would have been considered unreasonable and visionary in the extreme if not downright medical heresies. Conspicuous among these may be mentioned Wrights opsonic theory of injecting into the blood certain specific bacterins or serums that will so stimulate phagocytosis or the opsonic power of the white blood cells that they will become little corpuscular cannibals that will destroy and drive out every disease germ in sight and render the system absolutely sterile and immune against them.

So you see how essential it has become in these days of change and rapid scientific thought and discovery that physicians should keep themselves thoroughly informed on all physiological and biological subjects and especially those that pertain to the circulation of the blood, bacteriology, and its most powerful ally, serum therapy. The blood being the principal medium through which and into which the various bacteria toxines and other poisons must enter the system and find lodgment preparatory to begining their nefarious work of tissue poisoning and destruction, we should endeavor to learn some plan of preventing their entrance, of combatting their presence and off-setting their methods of proliferation.

We need to study their individual characteristics and learn their haunts and habits that we may be able with out antitoxins and blood serums to break into their strongholds, tear down their fortifications, and drive them from their entrenchments in the system; and at some time in the near future we expect to be able to do this in almost every case of germ infection by the application of appropriate bacterial serum just as we now do by innoculation with vaccine virus to destroy or counteract that certain pabulum, toxine or what not in the blood that feeds the germ of small pox. Now you would hardly expect a man to recognize a pathological condition in contra-distinction to a physiological one, if he were unfamiliar with the physiological. In order to understand and fully comprehend serum therapy one must familiarize himself with the different influences and agencies that conspire to bring about its peculiar manner of action. The importance of physicians keeping themselves at all times thoroughly informed on the circulation of the blood and its normal physiological functions together with a corresponding knowledge of bacteriology and pathology as an aid to a correct understanding of disease and the application of the serum treatment becomes evident.

Now, inasmuch as the food after digestion becomes absorbed by the lacteals and lymphatics and is carried by the portal or lesser circulation directly to the liver, the blood to that extent becomes an accessory of the digestive process for the ultimate purpose of nutrition and tissue building, which process is accomplished by the food elements being conveyed to the different parts of the body and tissues by the circulating blood current. Here we see what an important part the circulation plays as a carrier and the power it has any may exercise in the innoculation and spread of disease germs throughout the human system. Should they enter the system by the stomach, they are carried by the portal circulation to the liver and systemic circulation through which not only materials for repair are conveyed, but disease germs or curative agents as well, by being taken up by ingestion and absorption along with the food and carried through the portal circulation to the systemic circulation and tissues at large.

So we see while the portal circulation is primarily a carrier of nutrition, it may also become the purveyor of deadly disease germs or be utilized for the better purpose of conveying curative agents in the form of antitoxins. It is this phase of the circulation that we wish to study and strive to better understand and learn to more frequently utilize as a carrier of antitoxins and medicaments to the diseased tissues which in combination with the ingested and digested food elements are transported directly to the liver. Leaving the liver, it then goes to the right side of the heart with its normal constituents and toxins or antitoxins, thence to the lungs performing again the same office and in addition given off some of its poisonous gases for oxygen. Thence, it goes to the left side of the heart where together with its death-dealing toxins or its life-giving nutriment and medicaments, it is poured into the general systemic or arterial circulation either to poison or to purify the whole life giving stream. So you can easily see how bacteria and disease germs and their antagonistic serums, whether entering through peripherial lesions or by ingestion and absorption, can be rapidly assimilated by the simple and normal process of the circulation of the blood, and how poisonous germs or medicaments that may enter or become injected into the circulating fluid, either from within or without may easily gain access to the cells and tissues of the entire body.

Oftimes, the tissues and cells of the body becomes so weakened and overpowered by toxins and disease germs that natures' unassisted forces are unable to properly police the system until her diminished and impaired opsonins are replenished and rendered adequate to the task of driving out these hordes of invading microbes that have entered it and caused natures' powers to be overthrown and the energy of her immunizing and opsonic forces to be lost; then it is that we may be able by an intelligent understanding of the blood and circulatory apparatus and the vulnerable points of microbic life, by the use of hypodermic or intra-venous medications and those which can be administered by ingestion reach and counteract or neutralize these invading toxins, ptomaines, and other poisons before they become fully established in the system.

For as we have already seen, medicaments as well as poisonous germs can be rapidly taken up by absorption from the digestive tract and passed along with the ingested and digested food to the portal circulation, and thence to the general circulation of the entire body either to the restoration and preservation of its health-giving functions or to the poisoning of the tissues and the destruction of vital processes. So you can readily see how the germs of anthrax, syphilis, tuberculosis, typhoid fever, pneumonia and all that horde of disease germs, after gaining entrance to the circulation, may rapidly disseminate themselves throughout the body.

Now when we remember that the blood makes a complete circuit of the whole body every twenty to thirty seconds, then we can begin to appreciate to some extent the danger and power of the blood current as a germ carrier and also obtain some idea of its importance as a disseminator of curative serums, when injected into its current to stimulate its opsonic forces to resist disease and keep our bodies to the standard of health This process will be accomplished in direct ratio to the opsonic index of the blood cells and in proportion as the tissues receive and contain a normal and sufficient supply of healthy blood in every part of her vessels or circulatory apparatus.

In this way, the requirements for the health of the organism will be met and the whole scheme of life and health will resolve itself into the one condition, that we keep the circulation of the blood throughout the different parts of the body continually active and the opsonins in the blood up to the normal standard of their working capacity. Whereas, should we fail to do this, the circulation will at once become impaired and sluggish, its vital functions altered, its opsonic powers diminished and inefficient to the dangerous extent that we must immediately multiply physiologically, artificially, or otherwise. These deficient opsonins may be increased by the injection of appropriate bacterial serum and in suitable quantities, restore to nature her lost physiological balance, and enable her to drive out and resist invading organisms.

Ever since the day Mayerhofer isolated the streptococcus pyogenes from the blood of a dead puerperal woman, and Pasteur produced living cultures of the same and demonstrated it to be the principal cause of puerperal sepsis, it has been the dream of thte profession to produce a serum with which the poisoned system woman's blood could be reached in some way either by direct injections or by ingestion and absorption through the stomach. I believe that Mulford & Co., and other makers of antitoxins and bacterins are experimenting in the right direction. And who knows ere another decade shall pass but that we shall make even greater conquests over this terrible scourge with blood serums and bacterins than we have in the past with cleanliness and antisepsis until the horrors of the puerperal state shall become a byword of the past?

I believe that right along this line of serum therapy, we have the richest unexplored field of medical science. It devolves upon us as physicians to develop it to the end that we must familiarize ourselves with and take advantage of every agency and circumstance that bears upon it; or that will in any wise aid us in our undertaking. Whether it be the circulation of the blood, chemistry, bacteriology, pathology, or any other closely allied subject, we want to study the hematolytic, as well as the antitoxic action of bacterial cultures on the human blood, since there is unquestionably a significient relation between them not yet thoroughly understood. We want to obtain a more definite knowledge of the powers and functions of the antibodies found in blood serum and get a better working knowledge of the germicidal properties of the normal circulating blood in order that we may gain a clearer insight in to the combining and resisting powers of healthy human serum.

We have already learned that there is great variation in the capacity of different normal systems for appropriating and assimilating immunity-giving serum. And we would also like to understand the manner by which the babe appropriates the mother's immune bodies in the milk and why it is that nature-furniished nutrment is supplanted and artificially prepared food substituted, so many infants wither and die like tender grass before a killing frost. By the acquisition of just this one item of knowledge we may be enabled to wonderfully lower the high mortality rate among bottled-fed infants. We want to look in at the open door that leads to a knowledge and explanation of the normal activity that controls cell multiplication and the process which occurs in the circulating blood called autolysis or self digestion of inflammatory exudates, and understand how the production of these agents or ferments are brought about and controlled by the system. In short, we want to study the circulation of the blood in all of its relations to serum therapy, and serum therapy in all its relation to the circulating blood, because the two are so closely related, interwoven, and interdependent that, to understand the one, we must necessarily understand the other. And the acquisition of a comprehensive knowledge of both will wonderfully aid us in grasping the thousand and one intricate and puzzling clinical phenomena connected with the blood and serum therapy.

